# [Retracted] Effect of HMGN2 on proliferation and apoptosis of MCF‑7 breast cancer cells

**DOI:** 10.3892/ol.2025.15005

**Published:** 2025-04-02

**Authors:** Bo Fan, Sifeng Shi, Xiaofei Shen, Xiaolong Yang, Na Liu, Guixia Wu, Xiaojuan Guo, Ning Huang

Oncol Lett 17: 1160–1166, 2019; DOI: 10.3892/ol.2018.9668

Following the publication of this paper, it was drawn to the Editor's attention by a concerned reader that certain of the Transwell cell migration assay data shown in Fig. 2A on p. 1162 were already featured in different form in an article written by different authors at different research institutes that had been submitted to the journal *OncoTarget*; moreover, there were indications that some of the cells in one of the affected data panels in the above submission had been duplicated or removed, comparing the data with the other submitted article. Following an independent investigation of the data in the office, it was found that a number of other figures in this paper also contained data that had already been submitted for publication in other journals.

Owing to the fact that the contentious data in the above article had already been submitted for publication prior to its submission to *Oncology Letters*, the Editor has decided that this paper should be retracted from the Journal. The authors were asked for an explanation to account for these concerns, but the Editorial Office did not receive a reply. The Editor apologizes to the readership for any inconvenience caused.

